# Transfer learning for versatile and training free high content screening analyses

**DOI:** 10.1038/s41598-023-49554-8

**Published:** 2023-12-18

**Authors:** Maxime Corbe, Gaëlle Boncompain, Franck Perez, Elaine Del Nery, Auguste Genovesio

**Affiliations:** 1 Computational Bioimaging and Bioinformatics, Institut de Biologie de l’Ecole Normale Supérieure, PSL University, 46 Rue d’Ulm, 75005 Paris, France; 2 Biophenics Laboratory, Department of Translational Research, Cell and Tissue Imaging Facility (PICT-IBiSA), Institut Curie, PSL Research University, 26 Rue d’Ulm, 75005 Paris, France; 3 Dynamics of Intra-Cellular Organization - UMR144, Institut Curie, PSL Research University, Paris, France

**Keywords:** Cellular imaging, Computational platforms and environments, Phenotypic screening, High-throughput screening, Data processing, High-throughput screening

## Abstract

High content screening (HCS) is a technology that automates cell biology experiments at large scale. A High Content Screen produces a high amount of microscopy images of cells under many conditions and requires that a dedicated image and data analysis workflow be designed for each assay to select hits. This heavy data analytic step remains challenging and has been recognized as one of the burdens hindering the adoption of HCS. In this work we propose a solution to hit selection by using transfer learning without additional training. A pretrained residual network is employed to encode each image of a screen into a discriminant representation. The deep features obtained are then corrected to account for well plate bias and misalignment. We then propose two training-free pipelines dedicated to the two main categories of HCS for compound selection: with or without positive control. When a positive control is available, it is used alongside the negative control to compute a linear discriminant axis, thus building a classifier without training. Once all samples are projected onto this axis, the conditions that best reproduce the positive control can be selected. When no positive control is available, the Mahalanobis distance is computed from each sample to the negative control distribution. The latter provides a metric to identify the conditions that alter the negative control’s cell phenotype. This metric is subsequently used to categorize hits through a clustering step. Given the lack of available ground truth in HCS, we provide a qualitative comparison of the results obtained using this approach with results obtained with handcrafted image analysis features for compounds and siRNA screens with or without control. Our results suggests that the fully automated and generic pipeline we propose offers a good alternative to handcrafted dedicated image analysis approaches. Furthermore, we demonstrate that this solution select conditions of interest that had not been identified using the primary dedicated analysis. Altogether, this approach provides a fully automated, reproducible, versatile and comprehensive alternative analysis solution for HCS encompassing compound-based or downregulation screens, with or without positive controls, without the need for training or cell detection, or the development of a dedicated image analysis workflow.

## Introduction

A high content screen (HCS) consists of an experiment where a large set of parallelized and miniaturized cell-based perturbations are performed using well plates and imaged by an automated microscope. Usually, the same cell culture is dispensed into wells and treated with a library of perturbants such as small chemical compounds, or genetic deregulators such as siRNAs to knock down gene expression. After image acquisition of all conditions, an image and data analysis pipeline is designed to extract relevant cell-based information, in order to identify a few perturbations of interest, named hits^1^. Advances in microscope and dispensing automation have enabled the rise of high content screening (HCS) over the last 15 years. HCS is powerful and has led to significant discoveries in basic research. It is also increasingly used in drug discovery^2–4^.

Nevertheless, HCS remains a cutting edge technology that is not readily accessible on any bench, because it also comprises some impediments that have been gradually identified^5^. The most important ones are undoubtedly the image and data analysis steps because microscopy image content can vastly vary from one given assay to the next. Therefore, most of the time a dedicated image analysis algorithm or pipeline must be developed or fine-tuned for each assay^6^. This handcrafted image analysis step often provides a specific measure, such as the nuclear cytoplasmic ratio for a fluorescent marker. It can also typically generate fifty to a few hundred different measurements for each cell, with a few million to hundreds of millions of cells in a single experiment. In some cases, measurements can be summarized by computing averages or median profiles per well, and this much-reduced data set is often considered for further analysis rather than single cell data. In our experience, even since the advent of the deep learning era, it has still been the case that a dedicated model has had to be developed or trained with any change of modality, fluorescent label or even task, which eventually means for each assay^7–9^. For instance, transfer learning was used to predict the mechanism of action of drugs, but it is a specific task that required fine tuned training^10,11^. Furthermore, positive controls required for supervised learning are not always available. In practice, many assays rely solely on one measurement such as nuclear translocation, receptor internalization or even the cell count^12^. Once such a measurement is effective and automatically assesses what is visible by eye (e.g. cell count), then the metric can be used without positive control, simply by selecting samples that fall outside the negative control distribution. Altogether, with or without positive control, HCS image and data analysis relies on dedicated developments for each assay, either of a handcrafted image analysis algorithm or through supervised training of a deep learning model. While designing a dedicated image analysis method for identifying drug-induced cell phenotypes is often a good approach for HCS, it is tedious and may not be systematically possible. Furthermore, the lack of automation of this specialized step represents an important roadblock in the democratization of HCS.

To address this issue we propose a simple and generic deep learning (DL) pipeline based on transfer learning (TL) in order to provide a unique image analysis block that can be used in a versatile way for any HCS campaign whose purpose is hits selections. Importantly, the method does not require extraction of handcrafted image features nor even a training step; it is flexible to the presence or the absence of positive controls and does not depend on any parameter setting. It can thus be straightforwardly used to analyze images from any HCS without needing adaptation. Due to the fact selected hits from a screen do depend on the aim of the assay that itself often dictates the data analysis strategy, there is no such thing as an absolute ground truth to evaluate an HCS data analysis method. Therefore, we subsequently applied this approach to screening campaigns that we had already performed over the last years at the Biophenics platform of the Curie Institute in Paris, France, and compared it with the results we had previously obtained with dedicated feature extraction-based analyses in order to provide a qualitative comparison and emphasize the pro and cons of both approaches. We showcase that TL offers a good alternative to HC with the compelling advantage of being fully automated.

## Results

### A transfer learning pipeline for high content screening

**Figure 1:**
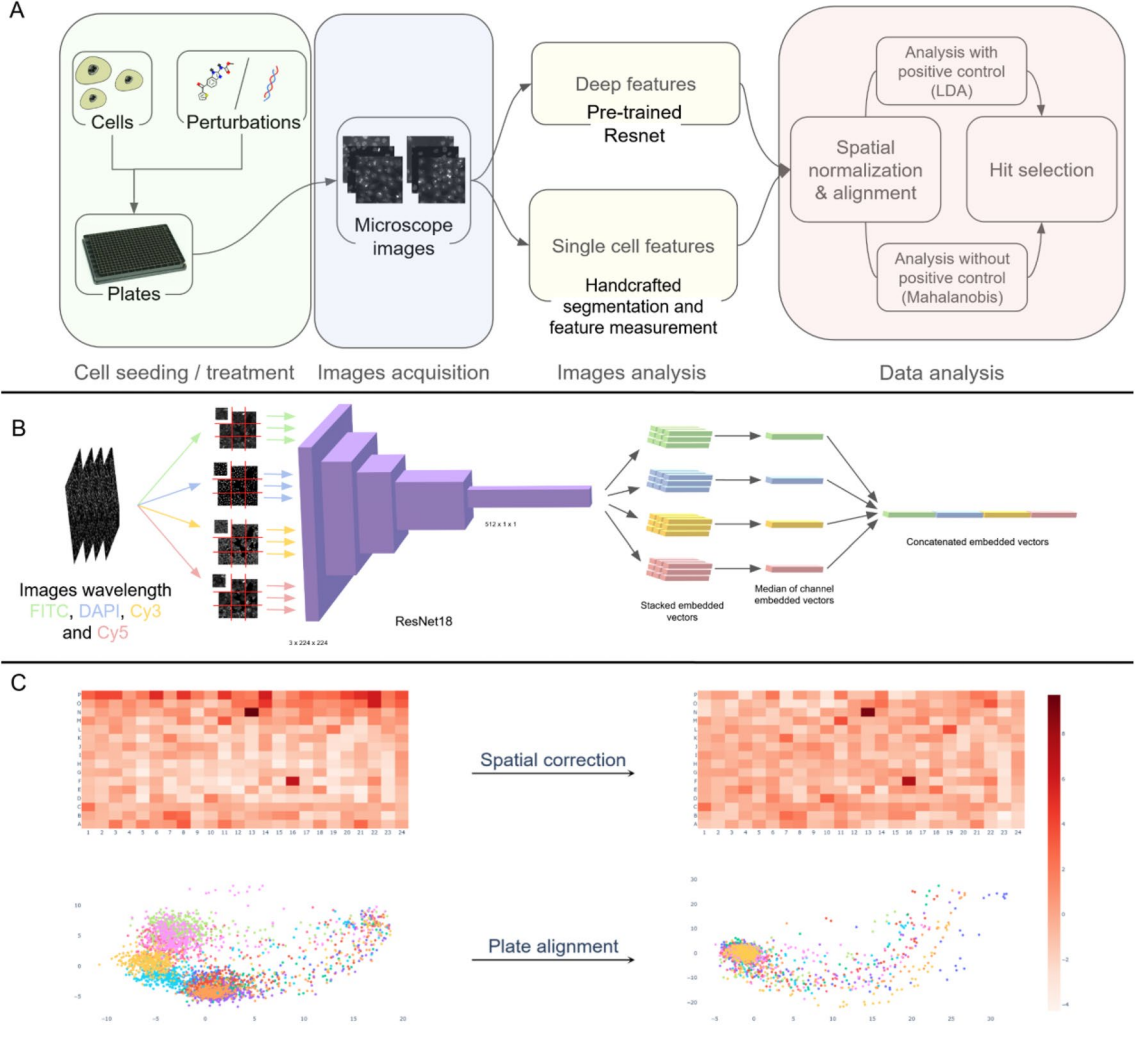
(**A**) Transfer learning (TL) and handcrafted (HC) analysis pipelines are exactly the same except for the image analysis part. Cell lines and compounds are plated into 384-well plates. Images are then acquired by an automated HCS microscope. For the TL features extraction, images are passed through a ResNet18 network pre-trained on Imagenet. For the HC feature extraction, an image analysis software (INcell Analyzer) is used to select or create a set of dedicated measurements. Both feature vectors are finally normalized the same way and a list of hits is selected following the same approach, either using a univariate feature directly or through a Linear Discriminant Analysis. (**B**) The automated image analysis pipeline is as follows: each image channel corresponding to a wavelength c C is split into tiles of 224 × 224 pixels then passed through a pretrained ResNet18. The median of the vectors collected this way for each tile is computed per channel. Finally the vectors for all c are concatenated into a single vector of 512 × c features for each image. (**C**) To observe the effect of plate correction and alignment a PCA is computed on all deep feature vectors of a screen (First principal axis to display the well plate heatmap correction and both the principal and second axes to display the effect of plate alignment). At the bottom each color represents a plate of the screen. A clear plate-effect splitting deep features of all plates apart can be corrected by the normalization process in the exact same way as it can correct plate effect on handcrafted features.

The typical HCS experimental and analysis pipeline is as follows. After cell seeding, drug treatment and cell labeling, images are acquired using an automated fluorescent microscopy platform. A dedicated assay-dependent image analysis algorithm, based on single cell segmentation, is then designed for automated extraction of quantitative features from each image. Then, single-cell phenotypic features are aggregated (mean or median) per each image field then per plate well. Following this, these profiles are spatially normalized and plates are aligned using each plate’s negative control distribution. Finally, using one or several extracted features, negative and optionally positive controls are used to select the phenotypic information of interest. The entire pipeline can be automated except the design of the image analysis workflow which requires dedicated developments relevant to the considered assay, cells, and image conditions. Here we describe how this step can be replaced by a fully-automated alternative which is assay- independent and doesn’t require any cell segmentation or training procedure (Fig. 1A).

ResNet18, a residual convolutional neural network (CNN) pre-trained on ImageNet^13^ was used to encode any microscopy image into a feature vector. To this end, we used a standard transfer learning strategy by removing only the last layer dedicated to classification. Furthermore, in order to make the encoder compatible with microscopy images with a variable number of color channels C, we tripled each image channel (the same information was repeated three times) to simulate one fake RGB image per image channel. Each of these RGB images was further spatially split to 224· × 224 pixels tiles to fit with the expected ResNet18 input. After processing, each of these tiles was then encoded into a 512 vector. Following this, a median vector was computed for all tiles for an image field, and all wavelengths of the same image field were concatenated into a single 512 × C vector. Finally, a median vector over all fields of a well was computed to obtain a single 512 × C feature vector per well (Fig. 1B). The output dataset was next treated as a standard list of handcrafted features obtained through a dedicated image analysis bloc and then corrected and normalized afterwards.

We first used a spatial correction procedure using a two-way median polish to mitigate positional effects^14,15^, followed by a plate alignment step using robust Z-score normalization on the negative control samples^1^. These post-processing steps ensure that all perturbations from any plate of a screen can be compared to another. Intriguingly, these corrections work just as well on handcrafted features as on deep features, correcting for the same effects both plate biases and plate misalignment (Fig. 1C).

In order to evaluate how closely such an automated pipeline would approach a handcrafted dedicated image analysis, we processed past screens from our platform and compared the results obtained to the actual hits that were previously selected. Screens were roughly divided into two categories, with or without a positive phenotypic control, based on the availability of control conditions used to depict expected phenotypic responses to perturbations.

### Transfer learning for HCS with a positive control

The goal of HCS data analysis is to identify perturbants with an expected phenotypic effect on a particular biological process of interest. We first tested the performance of our TL pipeline by applying it to an HCS where a positive control triggering the phenotype of interest was available.

For the regular handcrafted (HC) image analysis, single-cell features were extracted from four markers imaged in four channels using user-defined parameters to detect cell areas within the images (see Methods, positive control screens). An axis depicting the phenotypic differences between the negative and the positive control conditions was captured using a Linear Discriminant Analysis (LDA)^16^. This axis was then used to perform hit selection.

**Figure 2:**
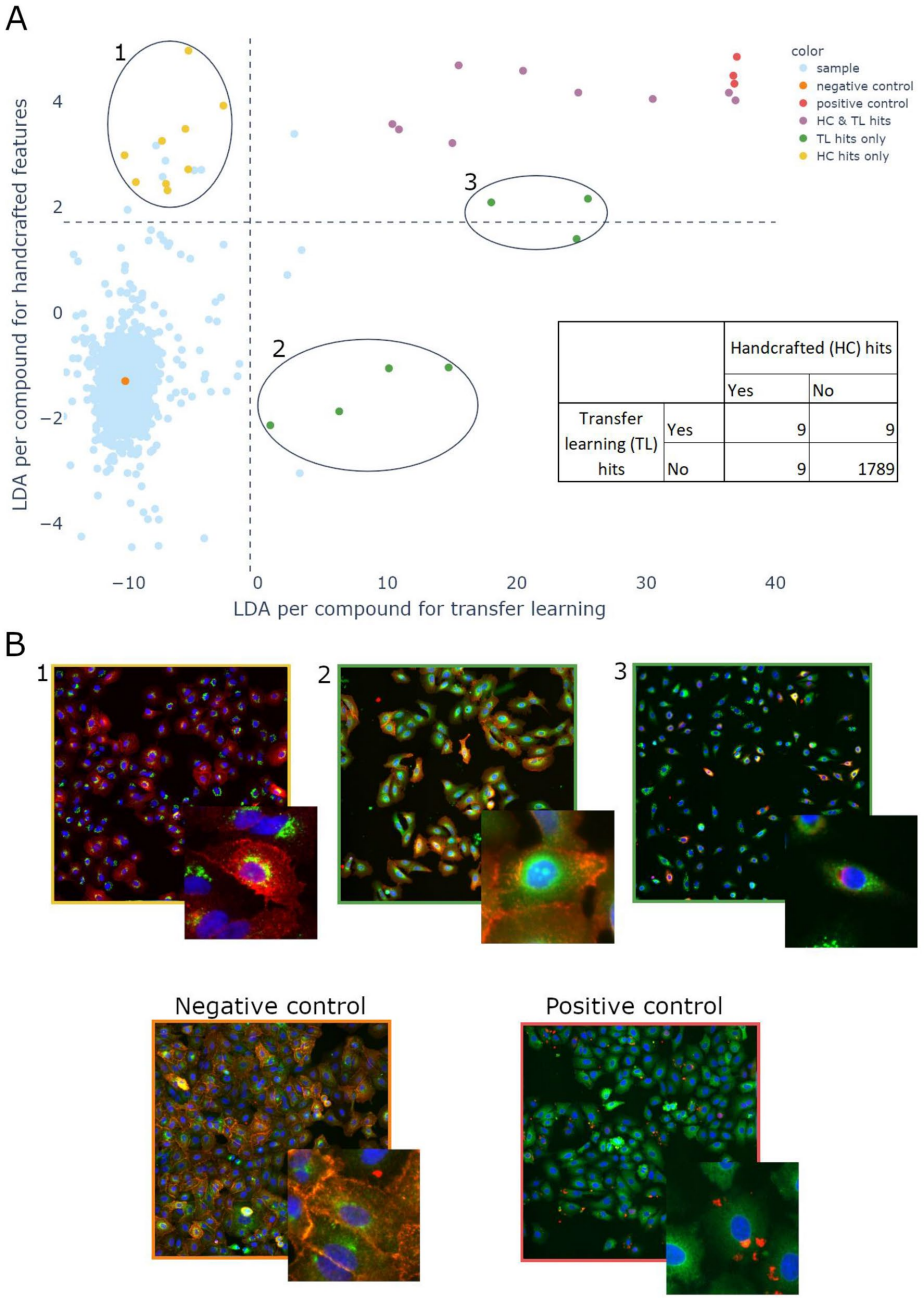
Transfer learning (TL) versus handcrafted (HC) analysis for a compounds screen with a positive control. (**A**) In both analyses, an LDA was performed between the negative control (orange) and the positive control (red). A threshold was then set on the LDA axis in order to select 1% of samples as “hits” (represented by the dashed lines). The color of the points depends on whether they have been depicted as hits in both analyses (violet), by the TL analysis only (green) or by the HC analysis only (yellow), or finally none of them (blue). The point displayed is the mean value of both replicates. However, both replicates must be above the threshold to be considered a hit. This explains why sometimes the point is above the threshold but not considered a hit. (**B**) Representative images of negative and positive controls as well as selected hits found in the HC analysis only (Group 1) or the TL analysis only (Groupe 2 and 3).

The TL pipeline was similar, except that the features were obtained as described in the previous section. A threshold was then applied on the discriminant axis to select approximately 1% of the screen as “hits”. Figure 2A displays the comparison between both approaches. Both methods agreed on the selection of the strongest hits (9/18), 9 hits obtained by the handcrafted analysis could not be identified as hits by the transfer learning approach. Reversely, the transfer learning approach selected 9 additional compounds as potential hits (2 not visible in the plot range due to the saturation of fluorescence). Among these, 4 were not identified by the handcrafted analysis and 3 were found very close to the threshold. As the hits previously selected with the HC analysis could not be considered “ground truth”, we pursued with a qualitative analysis by displaying representative pictures from 3 subsets of compound where the two analyses disagreed (Fig. 2B). As the exemples suggest, images in group 1, that contained compounds selected by the HC analysis but not by the TL analysis, didn’t look like the positive control. Reversely, the strongest hits detected by TL but not by HC (group 3) seemed very similar to the positive control. Altogether, these results suggested that about half of the hits were found by both approaches and that TL favored the selection of hits reproducing images close to the positive control compared to HC as assessed by eye.

The same comparative analysis could also be performed with the same pipeline on a siRNA screen with similar conclusions, showing that the approach seems to be effective for vastly different kinds of screens (see Sup. Fig. S1).

### Transfer learning for HCS without positive control

**Figure 3:**
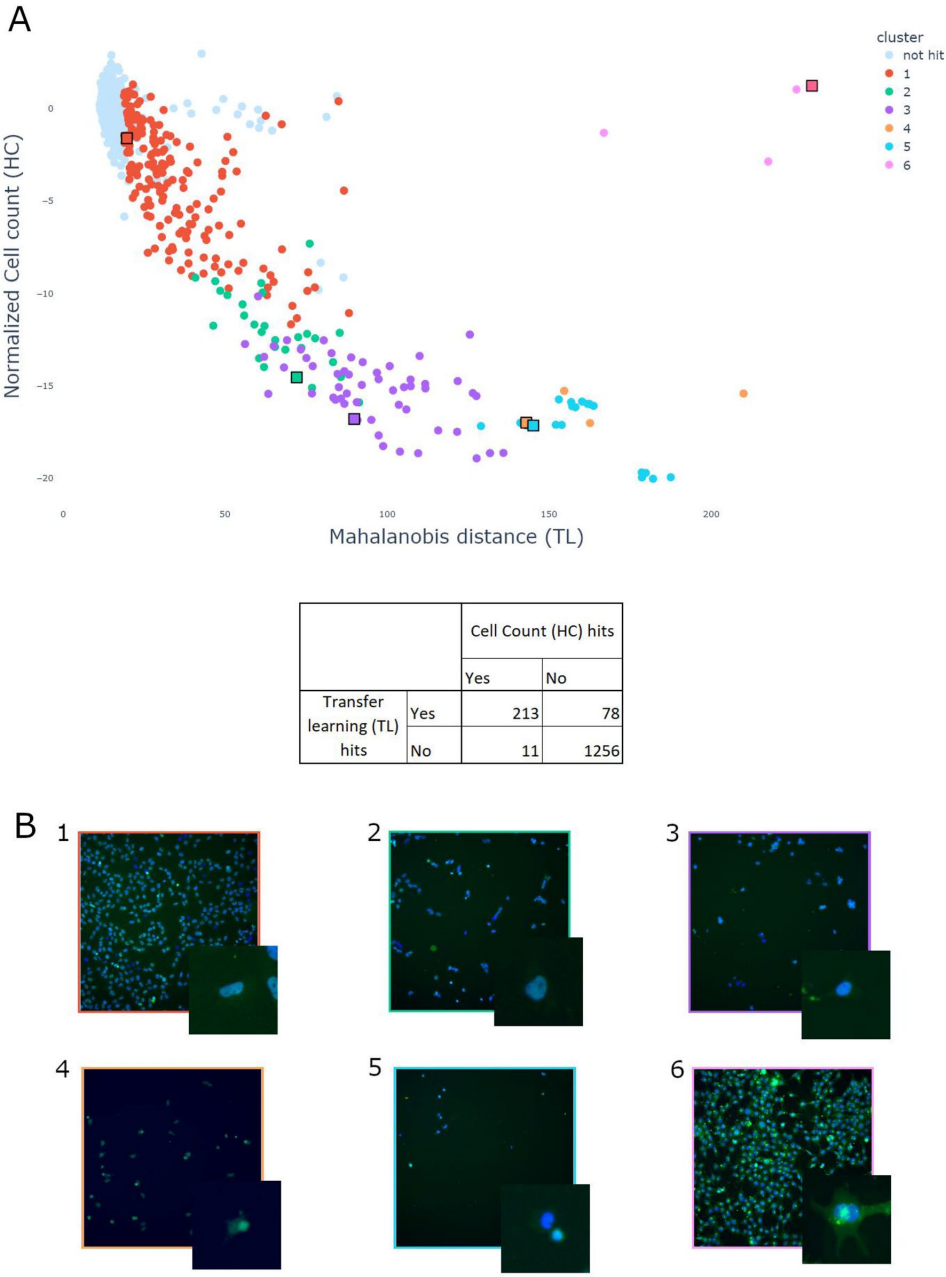
(**A**) Transfer learning (TL) versus handcrafted (HC) feature analyses for a compound screen without positive control. (**A**) Non-hits samples are colored in light blue and identified hits as out of the negative control distribution using TL are colored following 6 arbitrary groups defined by a hierarchical classification for easier interpretation. Each color indicates a cluster and each black square pinpoints the compound that is closest to the cluster center. (**B**) The image corresponding to each group center is displayed (1–6). Visualizing images grouped by close phenotypes is a good solution to further describe the distribution of hits. It complements the Mahalanobis distance that indicates only to what extent a condition is far from the negative control distribution. Interestingly, while cluster 1–5 seem to describe a decreasing cell count, cluster 6 seems to be located elsewhere in the feature space and describes a different phenotype present in only 3 compounds.

In the previous section we saw that transfer learning could be a good solution to obtain hits using a positive control in a straightforward way, without going through the burden of designing a dedicated handcrafted image analysis or even training a deep network. However, it is fairly common that positive controls are not available for an assay. On the Biophenics platform, we performed many screens without positive control. In this case, the distribution of a handcrafted measure over all the compounds or using the compound solvent wells (in general DMSO or water, aka negative control) is used to select hits as the compounds producing the largest effect for that measurement. The measurement of the considered feature has to be carefully designed as corresponding to a desired effect. It can also end up being very simple. For instance, cell count is one such feature that clearly measures cell death and can be used this way if, for instance, one seeks toxic compounds. In order to fully automate the analysis of an image based screen without positive control, we used the Mahalanobis distance to the negative control in the feature space obtained by transfer learning^17^. Similarly, after converting each image to a vector using a pretrained ResNet18, aggregating vectors over a sample well, normalizing features and aligning plates, we computed the Mahalanobis distance from all compounds to the negative control distribution. The rationale is that the largest distance to the negative control distribution should reflect the largest phenotypic difference to the untreated cell, thus the one most perturbed by the compounds. We then compared the resulting hits obtained with a dedicated cell count to this approach and report results Fig. 3. In the case of this assay, the Mahalanobis distance correlated very well with cell count, indicating that the main effect was cell toxicity.

Notably, the Mahalanobis distance did not correlate well with cell count for a few compounds, which indicated that while their Mahalanobis distance was high, in this case it was not in the direction of the cell count in the feature space and denoted another phenotype. We then thought that a good complement to the distance itself would be simply to arbitrarily cluster in feature space all the compounds that fell above a significant distance regarding the negative compound distribution, in order to be able to visually investigate rough categories of hits that fall outside of the negative control distribution. The result of such a clustering is reported Fig. 3. As expected, the clusters in the branch that correlated with cell count displayed a decreasing number of cells (cluster 1–5). However this approach also highlighted a cluster of 3 compounds (cluster 6) that could not have been selected by the handcrafted analysis (because their cell count is similar to the negative control), and displayed a completely different phenotype.

Furthermore, when applying this analysis on a screen for which we have (but fain to ignore) positive controls, we could demonstrate that not only were the positive controls selected as hits but also that a good amount of hits, selected with the approach using positive controls would have also been identified in this way (see Sup. Fig. S2).

We also evaluated specific channel images-based effects (see Sup. Fig. S3) and while most of the data correlated well between channels, we could observe particular effects (greater distance) for cluster 6 in the cell channel (wave 2). In particular, this approach performed well in identifying a few images of brighter appearance (cluster 6) when compared to the negative control images (cluster 1). From a visual standpoint, such compounds could be depicted as phenotypically interesting hits perturbing the endogenous cell fluorescence.

## Discussion

In this work, we propose a versatile training-free approach based on transfer learning to obtain hits from an HCS without going through the burden of designing a dedicated handcrafted image analysis which can be tedious and sometimes impossible with cells such as neurons that are difficult to segment. Once all images are encoded into feature vectors, thanks to a residual network pre-trained on ImageNet, our approach can be declined for screens with or without positive control. When a positive control is available, we use it alongside the negative control to project all compounds on a linear discriminant axis. We then use this axis to select conditions that best reproduce the positive control phenotype. When no positive control is available, we compute the Mahalanobis distance from the negative control as a measure of the amplitude of the cell phenotype change with respect to the negative control. We can then further categorize these hits in groups of phenotypes. In both cases, we show that the hits obtained by a handcrafted image analysis could be reproduced and even enriched.

It should be noted that when no positive controls are available, a handcrafted image analysis enables one to focus on a specific measure of interest such as nuclear translocation or cell death. Thanks to this focus, this approach could possibly be more robust than transfer learning in cases where a specific cell phenotype is sought but the cell count could be vastly different than on the positive control. On the contrary, the automated analysis approach we suggest will provide a metric to identify all phenotypic changes found in the screen without favoring one feature over the others. From this point, clustering can help to further compress these results to a handful of representative images that can be inspected in order to identify compounds of interest. While searching for a specific phenotype, this capability could be seen as less efficient, however, it is still fully automated compared to a user-guided HCS workflow that one needs to build. Furthermore, we have shown in this work that this approach can detect unexpected phenotypic changes that were not previously identified when focusing on particular handcrafted measurement.

A residual network, pre-trained on ImageNet, captures a powerful combination of low level features quantification that can be very useful in discriminating many types of images including microscopy ones. While we leverage on this capability, such encoders may possibly miss very subtle phenotypic changes. However, we have not experienced this so far while comparing results obtained by an handcrafted image analysis. As we have seen, it could also be that handcrafted image analyzes present limitations in detecting subtle or unexpected phenotypes. In any case, the results shown in this work could be reproduced on other compound-based or downregulation assays, and so far we have not witnessed any case where this would work less well than a handcrafted image analysis, suggesting that this approach should be robust for many types of assays.

Altogether, the transfer learning pipeline for HCS analysis which we propose is fully automated, can be used even when cells cannot be segmented, is efficient at selecting relevant hits, is fully reproducible, can be used for compound based as well as knock down screens, can be applied with or without positive control, and does not require developping a dedicated image analysis workflow. It provides a one fit-all analytic solution that should help democratize HCS.

## Methods

### Datasets

Screening data were collected from phenotypic ComPound Drug Screening campaigns (CPDS) screened in-house (BioPhenics platform, Institut Curie) using the Prestwick compound library (1280 off-patent small molecules, mostly approved drugs from FDA, EMA, and other agencies).

For the CPDS-negative screen, we employed A-673 cells (ATCC, CRL-1598) with compounds (10 µM) being incubated for 24 h, as previously described^18^. Image acquisition was performed using the INCell 2200 automated wide-field microscopy system (GE Healthcare) at 10× magnification (Nikon 10×/0.45), using the same exposure time for all plates in the experiment and across replicate experiments. Each well of the 384-well plates (wp) was imaged as an image set of two channels with four fields per well being collected for nuclei (DAPI wave 1) and cell channels (wave 2, endogenous tdTomato labeled protein) The screening was performed in duplicates with 24,576 images acquired per replicate experiment.

For positive control screens (CPDS-positive) we used A549 cells (ATCC, CCL-185) provided by F. Perez and G. Boncompain (UMR 144, Institut Curie) stably expressing a GFP-tagged RUSH reporter and a hook protein that allow a biotin-induced synchronous release of secretory cargos from the endoplasmic reticulum in a population of cells^19,20^. Briefly, the GFP-fused cargo is retained in the endoplasmic reticulum (ER) through a streptavidin-based interaction which is then relieved by biotin addition to cells. Cells were seeded in 384-wp (Viewplate 384, Perkin Elmer) for 24 h, treated with compounds at 10 µM for 90 min, then subsequently treated with 40 μM of biotin for 60 min. Brefeldin A-treated wells (BFA, Positive control) are used as positive control of GFP-cargo retention in the ER, whereas wells treated with DMSO alone are used as negative phenotypic control of GFP-cargo trafficking (Negative control). Image acquisition was performed using the INCell 6500HS automated widefield system (Molecular Devices, UK) at a 20× magnification (Nikon 20×/0.45, Plan Apo, CFI/60). Images of 4 fields/well were acquired for Hoechst 33,342 that stains the nuclei of cells (wave 1), GFP-fused cargo inside the cells (wave 2), rhodamine phospholipid to label all cells (wave 3) and finally for detection of GFP-taggedcargo arrival at the cell surface by immunofluorescence (wave 4). A total of 6144 images were acquired per 384-wp, resulting in 36,864 images per replicate experiment.

For both CPDS, screens were performed in duplicates.

### Handcrafted image analysis

Handcrafted image analysis first detects individual cells (stained with DAPI or Hoechst 33,342) using the proprietary INCell Analyzer Workstation 3.7 software (GE Healthcare) top-hat transformation method in order to identify and separate individual cells In CPDS-negative screen, once cells are identified, the output masks are used to detect the nuclear tdTomato labeled protein in wave 2. Other than cell count intensity and nuclei area features were computed as mean values per well.

In CPDS-positive, image segmentation was performed across all 4 image channels using multi-target analysis segmentation. Following nuclei detection, fluorescent rhodamine phospholipid (Cy3 channel 3) and GFP-labeled cells (FITC channel 2) were segmented using a region growing method to extract cell-by-cell features, including cell intensity, cell/background intensity, cell area. Intracellular GFP spots were detected in the FITC channel and defined as inclusions for the purposes of detection and segmentation. Min and max inclusion sizes were manually defined based on representative inclusions obtained in wells treated with the drug nocodazole. Such values specify the lower and upper limits of a size range within which expected GFP inclusions must fall. Extracted features included organelles count, inclusion/cell intensity and inclusion background intensity. GFP-tagged cargo labeling at the cell surface (Cy5 channel 4) was detected using a copy of the object mask previously generated in channel 2.

Once the segmentation parameters were optimized to phenotypically distinguish DMSO (negative control) and BFA-treated (positive control) wells, we subsequently used the extracted features to train the algorithm to classify cells according to the GFP-tagged cargo localization. A threshold was used to filter GFP-positive cells as ‘gfp-released’ using a single cell intensity feature (Cy5 channel 4) considering that any cells with normal GFP trafficking exhibited higher fluorescence in that channel. Neither BFA or BFA-biotin treated wells exhibited this fluorescence and were therefore discriminated as ‘gfp retained’.

### Transfer learning

ResNet18 from PyTorch module was used with pretrained weights on ImageNet (https://download.pytorch.org/models/resnet18-f37072fd.pth). We removed the last layer normally used for classification. The image normalization performed on microscopy channels was the same as the one used during training (centered and rescaled using the mean and standard deviation). Each image field was split into N tiles of 224 × 224 pixels. Those N tiles were stacked in batches and passed through the network. The output shape was then N × 512. It was then aggregated (median) per image to obtain a single vector with 512 numerical values (also called features). The different wavelengths corresponding to the same location were stacked together (C × 512 features) and finally those vectors, each corresponding to one image field, were aggregated per well using median.

### Data analysis

After feature extraction by handcrafted analysis or transfer learning, the data normalization and analysis pipeline was exactly the same in both cases. It includes a correction of plate spatial effects using a two-way median polish^14,15^ and the computation of a Robust Z score (RZscore) normalization^1^ to center and scale the plates. In case of multiple handcrafted features or in the case of transfer learning, a projection on the LDA or a Mahalanobis distance was determined based on whether or not a positive control was available. This single value computed per well could also be used to perform visual quality control by displaying plate-wise raw and transformed heat maps.

For identification of primary hits in the CPDS-negative handcrafted analysis, RZscores were calculated for each extracted feature. The median of the score of at least two independent replicate experiments was then used to score interesting wells. Only wells in which cell count, nucl/cell intensity or I × A features falls into the RZscore > 2 or <  − 2, in at least two replicate experiments, were labeled as “hits”.

For CPDS-positive, the same normalization was performed on four features : “Background Intensity”, “Cell Intensity”, “Cell/Bckg Intensity” and “GFP release”. Then LDA values were computed to select “hits” and an arbitrary threshold was chosen in order to retain 1% of compounds as hits.

In the deep learning workflow we used two different approaches depending on the availability or not of a positive screening control. Screen with positive control CPDS-positive: we used a Linear discriminant analysis^16^ to separate the distribution of points in two populations by taking a threshold along this newly created axis.

Screen without positive control (CPDS-negative): here we used Mahalanobis distance^17^, which allows us to compute, for each tested compound, a distance from the distribution of negative control. In the same way, we took a threshold distance value to separate into two populations. In this case, because we can not be certain of the real phenotype of those outliers, we have added another step, to classify all outliers into groups (hierarchical classification), to allow us to explore each group and the phenotype to which it is linked.

### Supplementary Information

Supplementary Information.

## Data Availability

Handcrafted and deep features computed from the large image datasets presented here can be made available upon request to elaine.del.nery@curie.fr or auguste.genovesio@ens.psl.eu.

## Code availability

The pipeline described in this manuscript is open and available online at https://github.com/biocompibens/BioPhenics_app along with some small example datasets. It can be easily used with any additional HCS dataset.
