# Supplementary material for: Transfer learning for versatile and training free high content screening analyses
Source: Sci Rep. 2023 Dec 18;13:22599. doi: 10.1038/s41598-023-49554-8 (PMC10730630; doi:10.1038/s41598-023-49554-8)
Supplement: Supplementary file 1 — Supplementary Information. [file 41598_2023_49554_MOESM1_ESM.docx]

# Supplementary figures


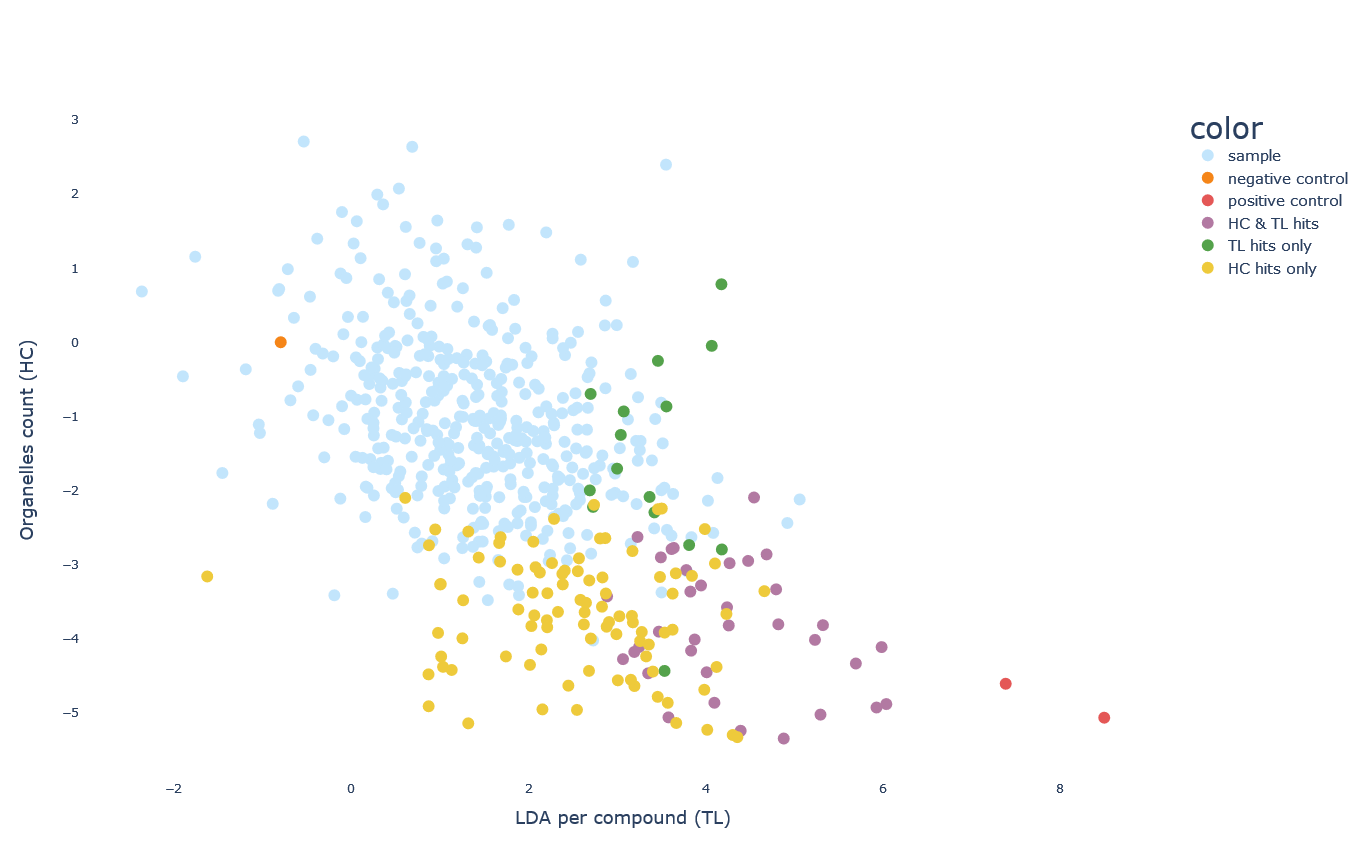


[**Supplementary figure 1**](#supfi_sirna): **Transfer learning versus handcrafted analyses for a siRNA screen with a positive controls.** The handcrafted feature is the mean number of organelles per cell in a well. In both analyses, plate normalization and alignment were applied to the data. For the handcrafted analysis a threshold was set at RZScore <-2 or >2 and for the transfer learning a LDA was computed with a threshold set as to retain ~10% of the siRNA with the largest effect. As four siRNA with different seed sequences were tested for each gene, a gene was selected as a hit only if 2 or more SiRNA were found above the threshold among these 10%.


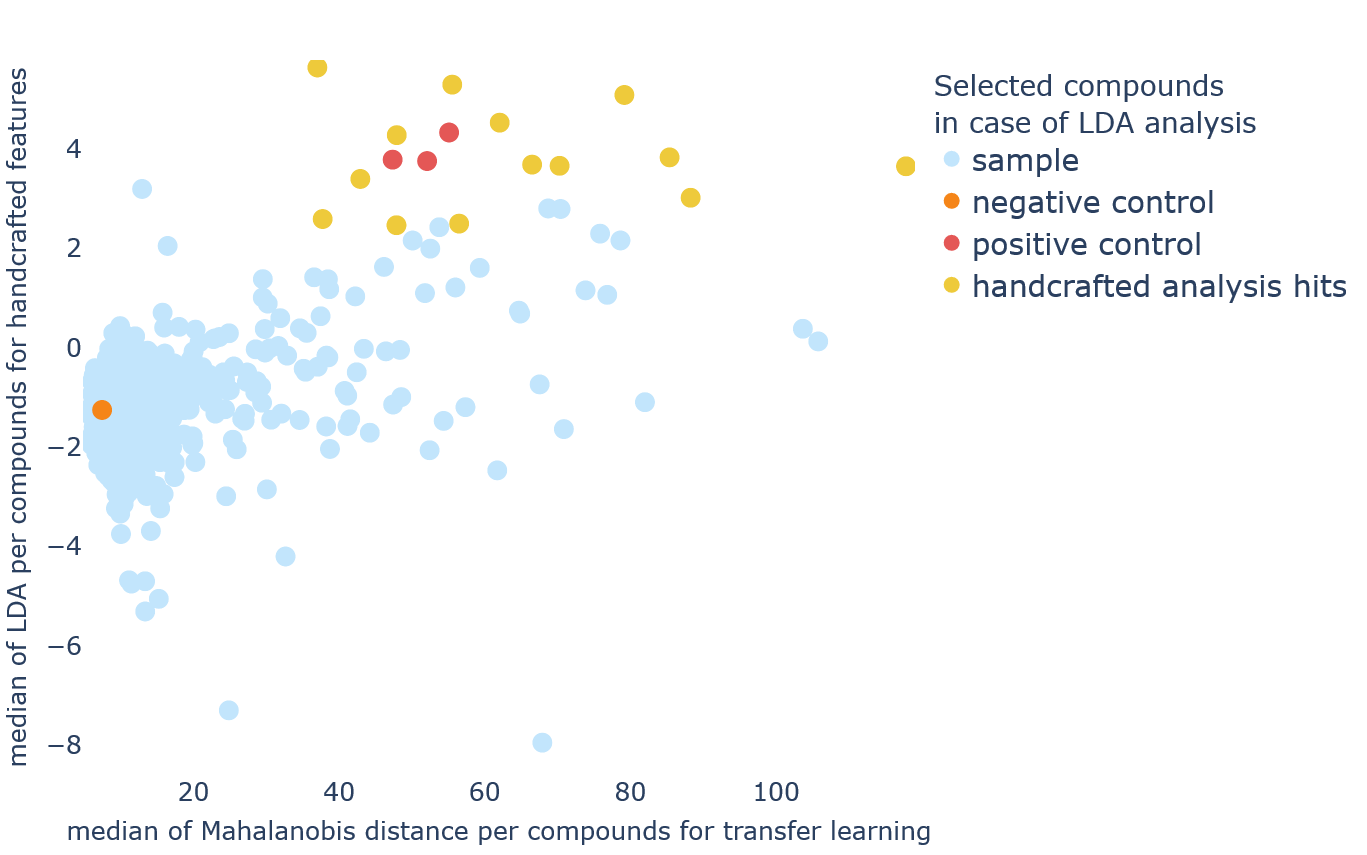
[**Supplementary figure 2**](#supfi_cpdspos): **Transfer learning versus handcrafted analyses for a compounds screen disregarding the positive control.** In these analyses, the positive control available for this screen was ignored and the strategy without positive control was applied. We then displayed where the positive controls (red) and the hits (yellow) obtained through the handcrafted analysis (considering positive control see [**Figure 2**](#fig_ace2compare)) would have been found with the transfer learning strategy. All hit and controls behind well on the left shows that the transfer learning strategy without positive control would enable us to still select the hits obtained using a handcrafted analysis with positive control. Furthermore, additional hits (blue dots on the right) would have been identified as other clusters displaying different phenotypes than the positive control (see [**Sup. fig. 5**](#sup_reddimneg) for details).

##


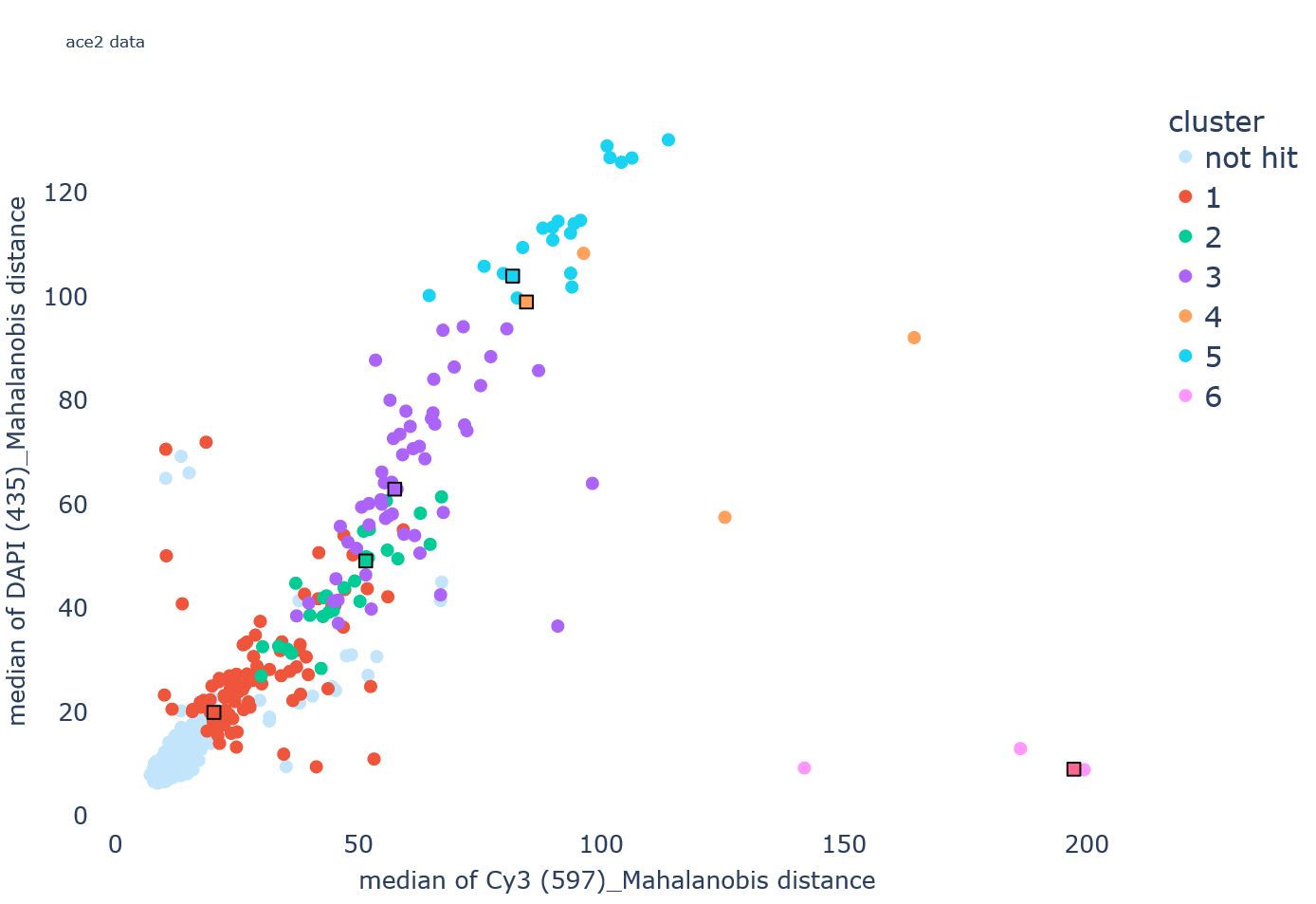
[**Supplementary figure 3**](#supfi_cpdsneg): Mahalanobis distance can be computed separately on each channel providing additional information. Here the Mahalanobis distance computed on TL features are plotted for the DAPI channel vs the Cy3 channel for the CPDS-negative screen. Both channels correlate rather strongly, indicating that they mostly provide the same information (cell count) except for the last cluster on the bottom right (6) that indicates a different phenotype in the Cy3 channel. The cluster ids correspond to those computed in [**Figure 3**](#fig_e45). For each cluster (=color) a black square frame pinpoints the compound that is closest to the cluster center. With this approach, cluster 6 is still identified as a different phenotype.


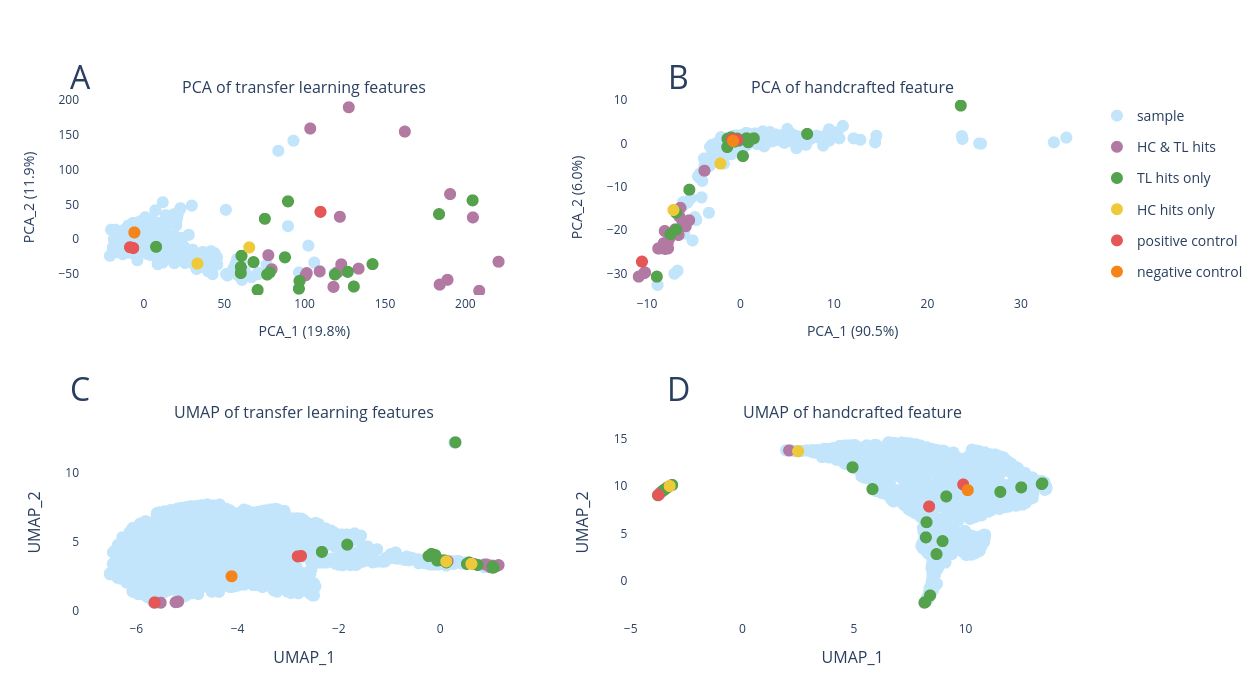


[**Supplementary figure 4**](#supfi_reddimpos): **Low dimensional projections of features in the HCS assay with a positive control presented in** [**Figure 2**](#fig_ace2compare)**.** A (resp. B) displays the two first principal components on the transfer learning (resp. handcrafted) features while C (resp. D) show two components UMAP on the transfer learning (resp handcrafted) features. With both TL and HC the main axis of variation of features does not align with the main axis but rather with the second. The LDA seems suited to easily obtain a classifier using the controls without training. UMAP transformation that requires parameter settings is less obvious to interpret


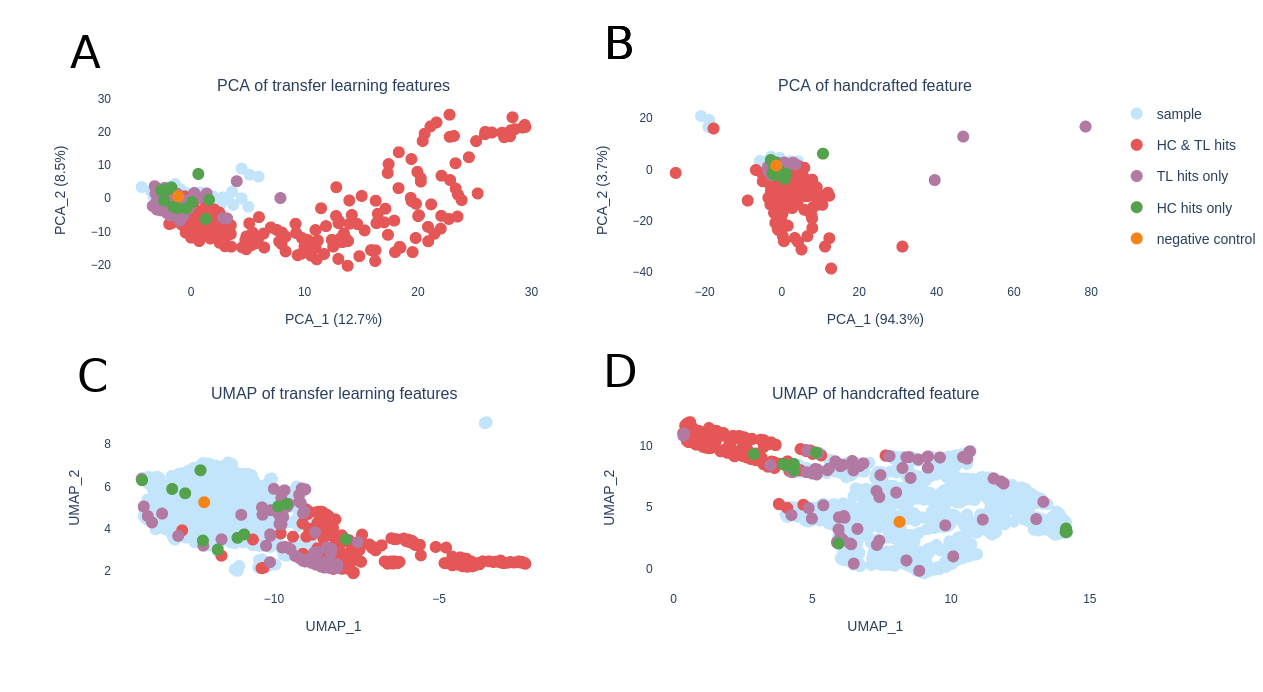


[**Supplementary figure 5**](#supfi_reddimneg) : Low dimensional projection of features in the HCS assay without positive control corresponding to [**Figure 3**](#fig_e45). A (resp. B) displays the two first principal components on transfer learning (resp. handcrafted) features while C (resp. D) show two components UMAP on transfer learning (resp handcrafted) features. In the four plots, hits seem located far from the negative control as expected by the large Mahalanobis distance criterium.


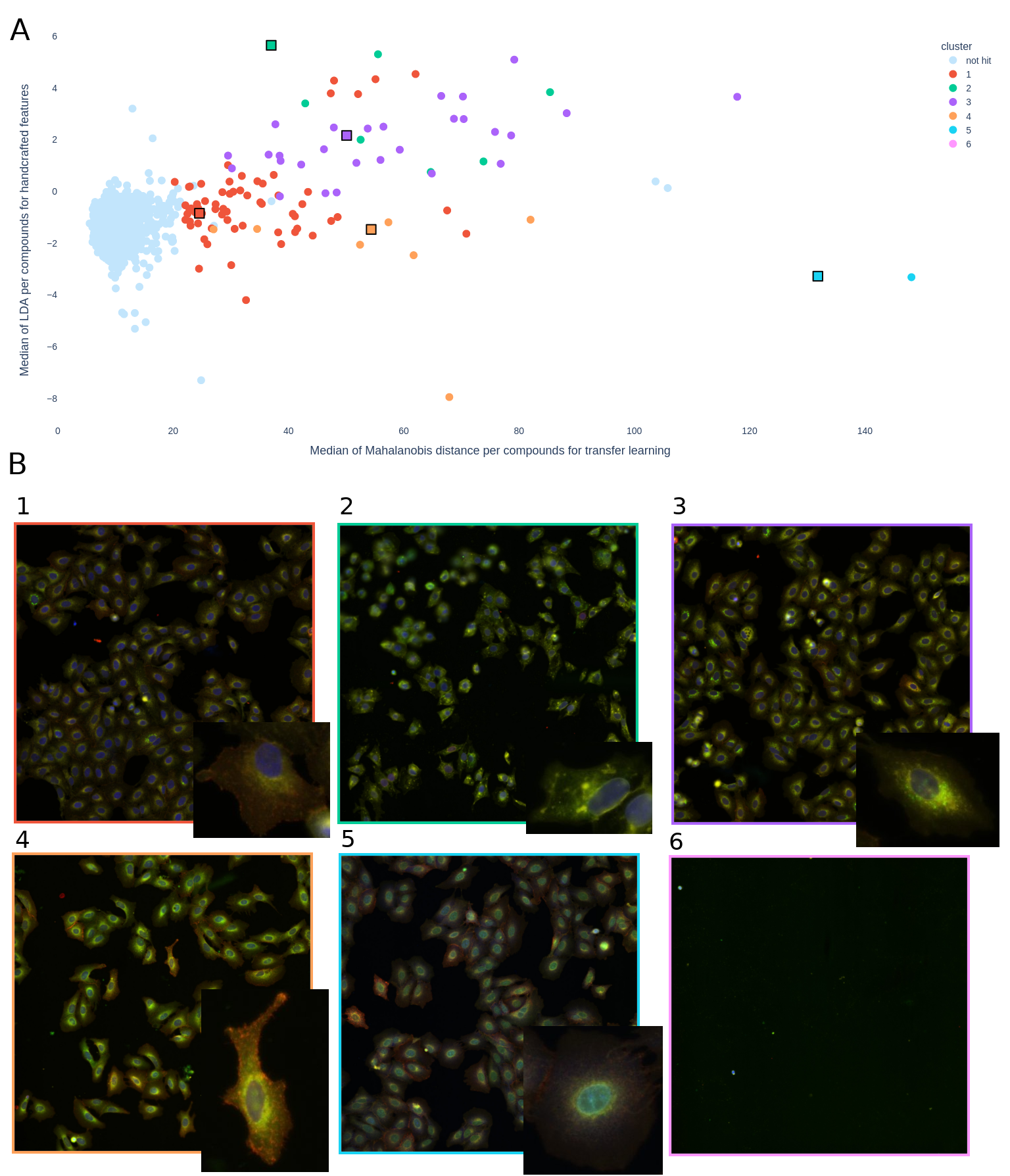


[**Supplementary figure 6**](#supfi_clusterwopos): A - K-means clustering in TL feature space of the datapoints displayed in [**Sup. fig. 2**](#sup_cpdspos). B - images of the closest compounds to each cluster center (square). Note that cluster 5 and 6 contain respectively 2 and 1 data points and the latter is located outside of the range displayed here.
